# Using a cohort study of diabetes and peripheral artery disease to compare logistic regression and machine learning via random forest modeling

**DOI:** 10.1186/s12874-022-01774-8

**Published:** 2022-11-23

**Authors:** Andrea M. Austin, Niveditta Ramkumar, Barbara Gladders, Jonathan A. Barnes, Mark A. Eid, Kayla O. Moore, Mark W. Feinberg, Mark A. Creager, Marc Bonaca, Philip P. Goodney

**Affiliations:** 1 The Dartmouth Institute for Health Policy and Clinical Practice, Geisel School of Medicine at Dartmouth, Hanover, NH USA; 2 Heart and Vascular Center, Dartmouth-Hitchcock Medical Center, Lebanon, NH 03756 USA; 3 Department of Medicine, Brigham and Women’s Hospital, Harvard Medical School, Cardiovascular Division, Boston, MA USA; 4 University of Colorado Medical Center, Denver, CO USA

**Keywords:** Random forest, machine learning, critical limb ischemia, diabetes, amputation, reintervention

## Abstract

**Background:**

This study illustrates the use of logistic regression and machine learning methods, specifically random forest models, in health services research by analyzing outcomes for a cohort of patients with concomitant peripheral artery disease and diabetes mellitus.

**Methods:**

Cohort study using fee-for-service Medicare beneficiaries in 2015 who were newly diagnosed with peripheral artery disease and diabetes mellitus. Exposure variables include whether patients received preventive measures in the 6 months following their index date: HbA1c test, foot exam, or vascular imaging study. Outcomes include any reintervention, lower extremity amputation, and death. We fit both logistic regression models as well as random forest models.

**Results:**

There were 88,898 fee-for-service Medicare beneficiaries diagnosed with peripheral artery disease and diabetes mellitus in our cohort. The rate of preventative treatments in the first six months following diagnosis were 52% (*n* = 45,971) with foot exams, 43% (*n* = 38,393) had vascular imaging, and 50% (*n* = 44,181) had an HbA1c test. The directionality of the influence for all covariates considered matched those results found with the random forest and logistic regression models. The most predictive covariate in each approach differs as determined by the t-statistics from logistic regression and variable importance (VI) in the random forest model. For amputation we see age 85 + (t = 53.17) urban-residing (VI = 83.42), and for death (t = 65.84, VI = 88.76) and reintervention (t = 34.40, VI = 81.22) both models indicate age is most predictive.

**Conclusions:**

The use of random forest models to analyze data and provide predictions for patients holds great potential in identifying modifiable patient-level and health-system factors and cohorts for increased surveillance and intervention to improve outcomes for patients. Random forests are incredibly high performing models with difficult interpretation most ideally suited for times when accurate prediction is most desirable and can be used in tandem with more common approaches to provide a more thorough analysis of observational data.

**Supplementary Information:**

The online version contains supplementary material available at 10.1186/s12874-022-01774-8.

## Background

Machine learning algorithms, such as random forest (RF) models, are an ensemble regression tree method commonly used for prediction and measuring variable importance in predicting an outcome of interest [1]. Ensemble learning methods use multiple learning algorithms, in this case the RF consists of multiple regression trees, to obtain better predictive performance than an individual algorithm. Machine learning algorithms have long been applied in computational research including data mining, artificial intelligence, and genomics [2–6]. In recent years, machine learning has been championed for use in health care research [7–9]. However, due to the lack of detailed understanding of machine learning algorithms among many healthcare workers, this method has been warily utilized among physician investigators where practical application and interpretability for clinical decision-making are essential.

### Logistic regression versus machine learning

The application of traditional regression approaches such as logistic regression as compared to machine learning approaches has been widely studied with no unqualified recommendation of which approach is best suited to analyzing data and making predictions [10, 11]. While the interpretability of logistic regression is considered an important feature, the predictive power of RF algorithms sway some in favor of its application. Multivariable regression approaches are frequently employed to produce prediction models. There is a growing consensus that machine learning and the advent of big data in health care offer new opportunities for understanding predictors of disease and treatment outcomes in medicine. Recent literature offers several new studies highlighting the application of RF in health services research, such as identifying multi-drug resistant tuberculosis (TB) versus drug-sensitive TB, predicting SARS COVID-19 infection, and predicting patients most likely to require dental implants. However, no studies to date have applied machine learning methods to examine predictors associated with outcomes for patients with peripheral arterial disease (PAD).

To fully understand the data at hand and how the end results of the analysis will be utilized, both approaches have their role in the comprehensive analysis of the data, particularly for prediction and understanding previously latent factors influencing outcomes. In this study we illustrate how the application of traditional generalized linear regression models and RF models can elucidate the role of sociodemographic and clinical covariates in predicting outcomes for patients with peripheral arterial disease and diabetes mellitus. This study is significant from past studies in that it leverages novel real world data sources, machine learning methods and multivariate logistic regression to provide complementary information for physicians caring for patients with PAD and diabetes.

### Using research on peripheral artery disease to illustrate identification of risk factors

Peripheral artery disease (PAD) results from a partial or complete buildup of atherosclerotic plaque in arteries carrying blood to the brain, limbs, and/or organs [12]. PAD occurs at the highest rates in patients with risk factors such as diabetes mellitus (DM) [12, 13]. where patients with both are at higher risk of amputation than those with either condition alone [14]. Furthermore, disparities in amputation rates have been observed among patients with concomitant lower extremity PAD and diabetes across race and geographic region [15–17]. Integrated management strategies for diabetes and PAD consist of preventative treatments such as hemoglobin A1c (HbA1c) testing, diabetic foot care, and vascular assessment with imaging. Several studies have shown that these interventions reduce amputation rates in patients with PAD and DM [18–25]. Effective implementation of the recommended integrated management strategies requires identifying the observable and modifiable risk factors which are most predictive of outcomes such as amputation and death in this population for targeted delivery.

### Goal of this manuscript

In this methodologic review, we use both traditional generalized linear regression models and practical approach to RF modeling to analyze the data and provide information on which sociodemographic and clinical covariates are most predictive of negative outcomes for patients with PAD and DM. By utilizing this approach we hope to provide insight into what observable factors influence the outcomes in this cohort of patients with PAD and DM. More importantly, we hope to provide a framework which outlines a more comprehensive analytic approach to applying traditional regression-based methods in tandem with interpretable machine learning techniques in order to provide the clearest insights in to risk adjustment for observational cardiovascular datasets.

## Methods

### Data sources and study population

We conducted an observational cohort study using a complete national sample of fee-for-service (FFS) Medicare beneficiaries in 2015 and 2016. We included all FFS patients who were newly diagnosed with concomitant peripheral artery disease and diabetes mellitus in 2015 and were United States residents between the ages of 65 and 95 at the time of diagnosis.

Their first claim in Medicare (the Physician Services/Carrier [Part B], Outpatient, or the Medicare Provider Analysis and Review [MedPAR] files) containing their first annual diabetes related International Classification of Diseases Ninth and Tenth Revisions (ICD-9 or ICD-10) code was used to define their index date. We also required patients to be FFS for 1 year following their index date to ensure we could observe their outcomes during the entire follow-up period. Additionally, patients had to have a diagnosis of an ulcer during the first 6 months of the index year so that we could ensure the ulcer occurred post diagnosis but before the outcomes of interest. Finally, patients had to be outcome-free (alive, no reinterventions or amputations) for at least 6 months following their index date so that the windows for exposure and outcome observation were distinct.

### Covariates

We gathered baseline health characteristics from the Medicare claims data, including patient-level comorbidities as determined by the individual disease indicators comprising the Charlson comorbidity index [26, 27]. We used the Medicare Master Beneficiary Summary File (MBSF) to determine each patient’s sex, race, age at diagnosis, Medicare-Medicaid dual-eligibility status, and used their ZIP code to determine whether they were rural or urban residing [28]. Our main clinical exposure variables include whether patients received at least one or more of the following integrated measures in the 6 months following their index date: HbA1c test, foot exam, or vascular imaging study.

### Outcome measures

We identified outcomes for the cohort in the 6 months following the exposure window and excluded any patients that had outcome events during the exposure window. Outcomes studied include any lower extremity amputation (both minor and major), any reintervention, and death. Death was determined from the Medicare MBSF while reintervention and amputation were determined using Current Procedure Terminology codes in the Part B and Outpatient files.

### Statistical analysis

We applied traditional statistical methods to describe the demographic and health characteristics of the cohort, including mean and standard deviations for continuous variables including age and comorbidity count, and counts and percentages for categorical variables (sex, race, exposures, Medicare-Medicaid dual-eligibility status, urban/rural indicator, and outcomes).

### Logistic regression analysis

We fit multivariable logistic regression models to assess statistical association between the outcomes of interest (amputation, death, and reintervention) and patient characteristics including demographics and comorbidities. Model-estimated odds ratios (ORs) and p-values with a significance level of 0.05 are presented. We compared the relative importance of each predictor in the logistic regression models using the absolute value of the t-statistic for each model parameter. In traditional logistic regression, the t-statistic is the parameter estimate divided by the standard error; as the significance of the parameter estimate is based on the t distribution, often quantified by the p-value, the larger the t-statistic the more significant the predictor. The goodness-of-fit for each model was determined using the McFadden pseudo-R^2 ^[29]. A McFadden pseudo-R^2^value between 0.2 and 0.4 represents excellent fit, where values close to 0 represents weak fit [30].

### Random forest (RF) modeling

Random forest (RF) models are collections of prediction trees, wherein many trees form a “forest” which can be used to provide a large number of trees to divide data elements [1, 10, 31–34]. A prediction tree is a non-linear approach to modeling complex data, which partitions the covariates into optimal splits until it achieves partitions allowing for the most homogenous subnodes.

### How random forest models work

Random forest algorithms grow many prediction trees to create the forest. First, the algorithm selects a training set. In this analysis, we selected 2/3^rds^ of the data, to fit each tree. The remaining 1/3^rd^ of the data is used as a test set to calculate the out-of-bag (OOB) error, an unbiased estimate of the classification error as trees are added to the forest with each iteration. That is, we used the estimated model to predict the outcome for those observations in the test set (those not used to estimate the model) and compared it to the observed truth. The OOB error is then the proportion of the test set that the model predicts incorrectly. We used the randomForest library [35] in R to fit the RF algorithm to our data. See Additional file 1: Appendix Figs. 1 and 2 for a depiction of the algorithm, it’s associated parameters, and application in prediction.

**Fig. 1:**
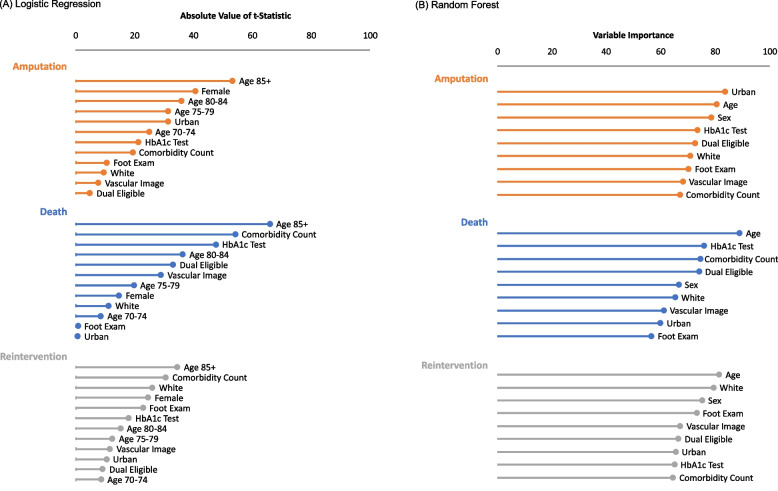
Variable importance by outcome from (**A**) logistic regression models and (**B**) averaged over the 500 iterations of the RF algorithm

**Fig. 2:**
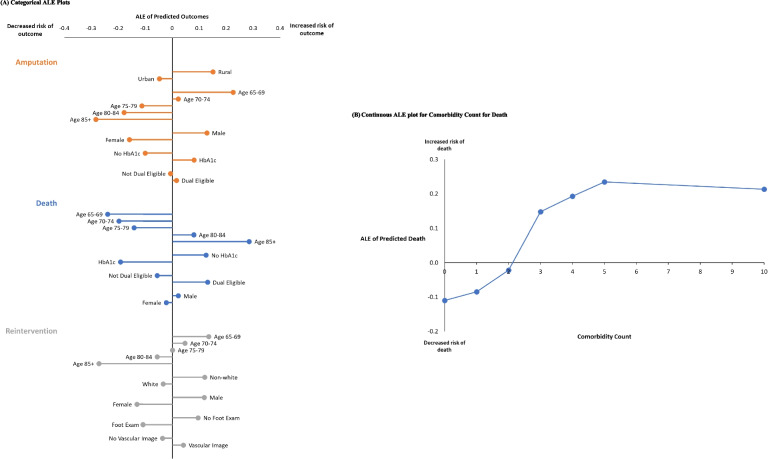
Accumulated Local Effects (ALE) plots for the top 5 most predictive covariates in the RF model fit to amputation, hospitalization, and death. **A** Categorical ALE Plots. **B** Continuous ALE plot for Comorbidity Count for Death

### Rare outcomes in RF modeling

A consideration in using a RF model is the use of an imbalanced classification dataset. That is, the observed outcomes in our dataset are rare events, thus the non-events are dominant in the dataset. If we fit the algorithm to the dataset as is, typically it would predict all observations in the test sample to have non-events, making the classification error appear small (equivalent to the event rate) and the resulting algorithm would appear to fit the data well while in reality, it would not identify any useful information in predicting events. With imbalanced datasets, the RF algorithm does not ascertain the necessary information about the rare events to make an accurate prediction. Hence, it is desirable to use RF algorithms with balanced data sets [36]. To fix the imbalance in the events, we performed a combination of oversampling (resampling patients with the event to balance the data) and under-sampling the non-events to obtain the same sample size as the original cohort. Both techniques use a random sampling algorithm to select cases to comprise the final analytic cohorts with balanced numbers of outcome events and non-events [37, 38].

### Variable importance in RF modeling

One key feature of RF models is their ability to produce a measure of variable importance. Variable importance in the RF models is estimated by examining how the prediction error increases when the data for each individual covariate is permuted while all other covariates are left fixed. This approach was repeated for each tree grown in the forest and then averaged over all trees. The difference in the number of correctly predicted outcomes in the variable-permuted test data from the number of correctly predicted cases in the original test data gives the variable importance for the individual tree. The average of this difference over all the trees in the forest is the raw importance score for each variable. This prediction error is calculated for each iteration of the algorithm, for each tree generated, and normalized by the standard deviation then averaged over all trees [1, 35].

### Variable effects in RF modeling

To describe how covariates influence the prediction ability of the random forest model, we use accumulated local effects (ALE) plots [39]. ALE plots describe how the covariates, commonly called features, influence the prediction in machine learning models, including RF models. For ALE plots, the cumulative effects of a given predictor value are calculated over a conditional distribution to quantify how the effect of a predictor on a target variable/outcome varies with the predictor’s value. The basic interpretation of an ALE plot is, conditional on the given value of a predictor, the relative effect of changing the feature on the prediction is the value given by the ALE plot, as all ALE plots are centered at zero (the sample means for the given predictor). Thus, the value for a given predictor on the ALE plot is interpreted as the difference from that covariate value to the sample mean prediction.

### Comparison of logistic regression and RF approaches

To compare the results of the RF analysis to more conventional regression-based methods, we iteratively estimated multivariable logistic regression analysis for each of the outcomes of interest on the training and test data in each iteration of the RF algorithm. As with the RF models, we controlled for age, sex, race, comorbidities, urban/rural indicator, Medicare-Medicaid dual eligibility status, and each of the exposures of interest (HbA1c, foot exam, and vascular imaging). We fit the model on the same over/under sampled data used for the RF algorithm. We produced the prediction error rate of the estimated logistic regression model by predicting each outcome from the estimated probability for each model on all patients in the test set using a probability cutoff of 0.5.

We compared this to the prediction error rate for the same data using the RF methodology. We also compared the variable importance rankings of the covariates as determined by the two methods. This comparison emphasizes the importance of the multipronged approach to analyzing the data for both associations (regression) and predictive importance (RF) in a cohort such as this where areas for intervention are unclear.

All analyses were performed using SAS version 9.4 (Cary, NC) and R version 3.6.1 [40]. This study was approved by our Institutional Review Board, the Dartmouth-Hitchcock Health Human Research Protection Program (STUDY00030829) with waiver of informed consent.

## Results

### Characteristics of the Cohort

From 2015, there were 88,898 FFS Medicare beneficiaries diagnosed with concurrent PAD and diabetes with an ulcer in our cohort (Table 1). In the original cohort, before over/under sampling, 25% (*n* = 22,235) of patients were Medicare-Medicaid dual-eligible in the year of their diabetes diagnosis. The majority of patients in the cohort were white (82%), female (53%), and urban residing (81%). The average age of the cohort was 76.6 years, with an average comorbidity count of 1.73 using the Charlson comorbidity index. The rate of preventative treatments for these patients in the first six months following diagnosis were 52% (*n* = 45,971) with foot exams, 43% (*n* = 38,393) had vascular imaging, and 50% (*n* = 44,181) had an HbA1c test. Finally, the rate of outcomes among those in the original cohort included a mortality rate of 4.5% (*n* = 3,977), a reintervention rate of 3.6% (*n* = 3,175), and an amputation rate of 1.6% (*n* = 1,407). The over/under sampling balanced the cases and non-cases for each outcome with the final analytic cohorts for each outcome given in Table 1.

**Table 1:** Cohort characteristics for patients with a concomitant diagnosis of PAD and diabetes with an ulcer in 2015, overall and after over/under sampling each outcome (*N* = 88,898)

Characteristics	Original Cohort		Over/Under Sampled Amputation Cohort		Over/Under Sampled Death Cohort		Over/Under Sampled Reintervention Cohort	
	**Mean/N**	**StdDev/%**	**Mean/N**	**StdDev/%**	**Mean/N**	**StdDev/%**	**Mean/N**	**StdDev/%**
**Age in years**								
65–69	22,981	26	30,615	34	17,346	20	26,677	30
70–74	18,257	21	19,027	21	15,173	17	19,456	22
75–79	15,405	17	14,556	16	13,975	16	15,355	17
80–84	13,639	15	11,585	13	14,851	17	13,006	15
85 +	18,616	21	13,115	15	27,553	31	14,404	16
**Dual Eligible**	22,235	25	23,440	26	26,287	30	22,371	25
**White**	73,125	82	71,669	81	73,812	83	69,908	79
**Female**	46,828	53	39,992	45	46,610	52	42,616	48
**Foot exam**	45,971	52	46,291	52	47,607	54	42,122	47
**Vascular Image**	38,393	43	38,089	43	41,946	47	40,594	46
**HbA1c**	44,181	50	48,853	55	35,303	40	47,797	54
**Urban**	72,157	81	67,921	76	71,880	81	70,376	79
**Charlson Count**	1.73	1.69	1.85	1.69	2.08	1.84	1.93	1.78
**Death**	3,977	4.5			44,495	50		
**Amputation**	1,407	1.6	44,495	50				
**Reintervention**	3,175	3.6					44,495	50

### Logistics regression results

#### Variable effects and variation explained

We examined the association of the covariates with each of the outcomes using multivariable-adjusted logistic regression models (Table 2). We found for amputation all covariates were significant predictors. A patient being urban-residing had a protective effect against amputation (OR = 0.59; *p*-val < 0.001). The covariate with the largest odds of amputation is receipt of HbA1c testing (OR = 1.36; *p*-val < 0.001) followed closely by foot exam (OR = 1.16; *p*-val < 0.001). Vascular imaging decreases the odds of amputation (OR = 0.90; *p* < 0.001). Age is protective against amputation (for 85 + year olds vs. 65–69 OR = 0.29; *p*-val < 0.001) with the likelihood of amputation decreasing as age decreases. Results were similar for reintervention and death.

**Table 2:** Logistic regression results for models fit on the over/under-sampled cohorts for each outcome

Covariate	Amputation			Death			Reintervention		
	**OR**	**t-stat**	***p*-value**	**OR**	**t-stat**	***p*-value**	**OR**	**t-stat**	***p*-value**
**Foot Exam**	1.16	10.49	< 0.001	1.01	0.74	0.457	0.73	-22.84	< 0.001
**HbA1c**	1.36	21.23	< 0.001	0.49	-47.56	< 0.001	1.29	17.90	< 0.001
**Vascular Image**	0.90	-7.66	< 0.001	1.54	28.89	< 0.001	1.18	11.55	< 0.001
**Urban**	0.59	-31.33	< 0.001	1.01	0.62	0.534	0.84	-10.43	< 0.001
**Comorbidity Count**	1.09	19.27	< 0.001	1.25	54.12	< 0.001	1.13	30.49	< 0.001
**Dual Eligible**	1.09	4.80	< 0.001	1.76	33.05	< 0.001	0.86	-9.05	< 0.001
**White**	0.84	-9.54	< 0.001	1.26	11.18	< 0.001	0.62	-25.99	< 0.001
**Female**	0.56	-40.52	< 0.001	0.80	-14.69	< 0.001	0.71	-24.59	< 0.001
**Age in years**									
65–69	(reference)			(reference)			(reference)		
70–74	0.62	-24.85	< 0.001	1.23	8.42	< 0.001	0.85	-8.60	< 0.001
75–79	0.52	-31.36	< 0.001	1.63	19.67	< 0.001	0.77	-12.36	< 0.001
80–84	0.44	-35.87	< 0.001	2.45	36.31	< 0.001	0.71	-15.20	< 0.001
85 +	0.29	-53.17	< 0.001	4.42	65.84	< 0.001	0.46	-34.40	< 0.001

The McFadden R^2^ = 0.071 indicating approximately 7% of the total variation in amputation risk is explained by the model, falling outside the 0.2–0.4 range representing excellent model fit. For reintervention, the McFadden R^2^ = 0.046, which signifies less than 5% of the observed variation is explained by the model. Finally, the McFadden R^2^ = 0.124 for death, which means the model for death explains almost double the variation as explained for amputation and over double than explained for reintervention. Thus, the McFadden R^2^ values for each of the logistic regression models indicated that the chosen predictors did not explain a significant portion of the observed variance using these models.

#### Variable importance

Variable importance, as assessed by the absolute value of the t-statistic (Fig. 1a), tells us that age (85 + , t = -53.17), sex (t = -40.52), and urban/rural status (t = -31.33) are the most important predictors of amputation in the logistic regression model. While these factors were important, factors including dual eligibility status, receipt of a vascular image, and race are the least predictive of amputation. Similar findings for reintervention and death outcomes are shown as well (Fig. 1a).

### Random forest results

#### Variable importance

In Fig. 1b, we see the results of the variable importance calculations from the RF estimation for each of the 3 outcomes. For the prediction of amputation in the cohort, the most important variable is whether they are rural- or urban-residing. We see the level of importance for the other covariates including age and sex follow closely behind. The first clinical variable, HbA1c testing, is the fourth most important variable in the prediction of amputation followed by dual-eligibility. As with our logistic regression models, similar results are shown for reintervention and death.

#### Variable effects

In Fig. 2a, the ALE plot for amputation indicates that being rural-residing increases the likelihood of amputation compared to the mean population, while being urban-residing decreases it. Similarly, as age (category) increases, the less likely amputation is to occur compared to the mean population. Females are less likely than the mean population to receive an amputation, as are those who are not dual-eligible. Those not receiving an HbA1c test are also less likely than the mean population to receive an amputation.

For reintervention, certain differences were evident. The younger the patient, the more likely they are to have a reintervention compared to the mean population. Patients who are non-white are more likely to have a reintervention than the mean population while white patients are less likely. Finally, for death, we see the older the patient is, the more likely they are to die. Receiving an HbA1c test, being dual-eligible, and male all increase the likelihood of death compared to the mean population. In Fig. 2b, we see the ALE plot for death as comorbidity count varies. We see the ALE plot crosses zero at approximately 2, meaning those with 2 or fewer comorbidities are less likely to die than the general population and with those with greater than 2 comorbidities having a greater likelihood of death than the mean population.

### Comparison of logistic regression and random forest approaches

#### Variable importance: RF models can help highlight hidden themes

Assessing variable importance of each model produced similar results from each approach. The top 3 variables for each outcome in each model are the same, except for death where the logistic regression model indicated comorbidity count as a highly significant predictor, and where this predictor is ranked least important in the RF model, and sex was included in its place in the RF model, following a close 4^th^ in the logistic regression model. However, the variation in the variable importance measure in the RF models is noticeably less than in the logistic regression models. This indicates that the predictive power of each variable according to the RF model is much closer, highlighting the necessity for comprehensive analyses and importance of non-dominant subgroup analysis. For example, age (or at least 1 level of age in the logistic regression models) is the most significant predictor (or the second most) in both models for amputation, death, and reintervention. Notably, age is not the most significant predictor for the RF model for amputation. The sensitivity of the approach was able to tease out a key difference in amputation rates for rural- and urban-residing residence; the logistic regression model did identify this difference as well, following age and sex. This illustrates how the RF model can potentially tease out differences highlighting significant and non-dominant subpopulations for intervention or further study.

#### Variable effects: RF models and logistic regression often have similar effect sizes

For amputation, for the top 5 most predictive covariates determined in both techniques, we see exact concordance in the directionality of the conclusions. For example, in the RF model, rurality increases the risk of amputation compared to the general population, while the logistic regression results show a ratio less than 1 for urban vs. rural indicating rurality decreases the odds of amputation compared to urban-residing population.For death and reintervention, we see similar concordance among the directionality of conclusions according to the logistic regression results and the ALE plots for the RF models.

#### Predictive capabilities: RF models often illustrate lower error rates

**Fig. 3:**
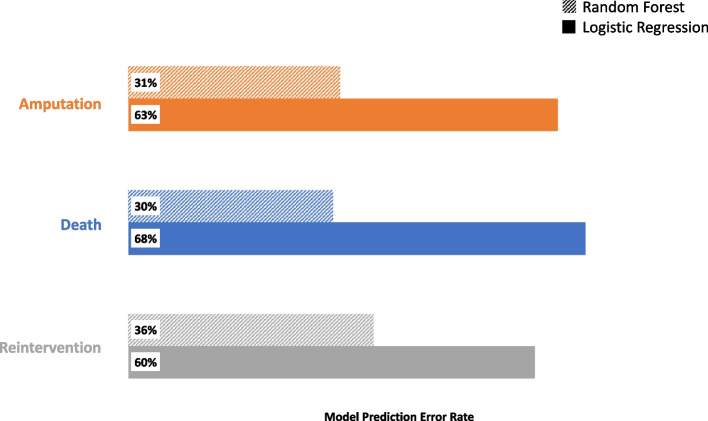
Average prediction error rates for the random forest (RF) and logistic regression models. RF prediction error rate is the out-of-bag (OOB) error rate. Logistic regression test sample prediction error rate calculated using average error rate across 500 iterations

Finally, in Fig. 3, we see the average results of prediction on the test sample created in each iteration of the RF algorithm. The OOB error rate, on average, when predicting amputation is 31%, while for the same test datasets is 63% using the estimated logistic regression models. Similarly, for reintervention the RF OOB error rate is 36% while for logistic regression it is 60%. For death, we see an OOB error rate of 30% while logistic regression has a prediction error rate of 68% when predicting for test data, averaged over 500 iterations.

## Discussion

In this study, we analyzed factors predicting amputation, death, and reintervention in a cohort of patients with peripheral artery disease and diabetes mellitus, and an ulcer using both a logistic regression approach and a random forest approach. In general, both approaches provided similar results. However, the RF approach illustrated differences in certain subgroups, highlighting significant and non-dominant subpopulations for intervention or further study. Specifically, in the RF approach, we found patient age 65–69 was most predictive of amputation, as shown on the categorical ALE plots in Fig. 2, but whether the patient is rural- or urban-residing is the most predictive observed covariate (Fig. 1b). In other words, though urban/rural status is the most important predictor of amputation, and being rural leads to an increased risk of amputation compared to being urban, being in the youngest age group confers the largest single increased risk of amputation. In the RF model, HbA1c testing and comorbidity count follow age in the most predictive covariates of death in this cohort, where the HbA1c test is given more often in those who die, a consequence of healthy patients not receiving monitoring as closely as those symptomatic patients, and the likelihood of death increases with the patient’s comorbidities.

### Using advanced statistical methods to complement common approaches

RF algorithms have a number of statistical and computational strengths. RFs are versatile in terms of the structure and types of data analyzed including both regression and classification approaches to estimation [35]. The algorithms use of only a subset of features at a time allow it to process significantly faster than other machine learning algorithms and ultimately allows for rapid training and prediction across many trees. Additionally, unlike typical regression approaches including logistic regression, RFs are robust to outliers with their predictive power little influenced [41]. Finally, each tree in the RF has high variance and low bias. Averaging across many such trees to create the final RF model results in a final model with low bias and only moderate variance [42].

While the relationship between the outcomes and the covariates given by the logistic regression results and the random forest models are similar in directionality, the RF results offer two key advantages to the regression approach. First, the RF approach offers a substantive and sensitive approach to identifying which covariates are most predictive and have the greatest importance in predicting the outcomes of interest. Second, the RF models offered increased predictive power over the classic logistic regression while using the same covariates. In fact, the prediction error rate was twice as high in logistic regression models than in the RF models. These two properties, when paired with the common interpretability of the logistic regression approach, provide a framework for analyzing data in a population with clear magnitude of associations between the outcomes and covariates (regression) and the ability to deeply analyze which covariates are most predictive of the outcome and best suited for clinical consideration and/or intervention (RF). Potential extensions to the current analysis would be to include more clinical factors in the RF model to increase the predictability and more modifiable patient or care patterns to potentially prevent or delay negative outcomes for this cohort.

On the other hand, there are some limitations to using RF models. While RFs excel at classification, they do not predict beyond the range of the covariates in the training data. Additionally, RFs may overfit datasets that are particularly noisy, that is data with an abundance of unexplained variation. This is potentially problematic in healthcare research where we are limited by the information in the Medicare claims data. The use of over/under sampling adds a layer of complexity and data manipulation that many audiences may not be familiar or comfortable with. It overemphasizes cases (in the current study), stressing the influence of covariates that may ultimately impact a small portion of the true sample population. Finally, the lack of understanding of random forest plots among clinical audiences, may make interpretation and practical application in health care settings less likely. Thus, utilizing RF models in tandem with traditional regression approaches may best serve to establish the use of RF models in healthcare research while also improving the ability to predict potential outcomes and pathways for patients.

## Conclusion

The use of RF models to analyze data and provide predictions for patients holds great potential in identifying modifiable patient-level and health-system factors and cohorts for increased surveillance and intervention to improve outcomes for patients. RFs are incredibly high performing models with difficult interpretation most ideally suited for times when accurate prediction is most desirable and can be used in tandem with more typical methods and tools (ALE plots, logistic regression) to provide a more thorough analysis of observational data.

## Supplementary Information

**Additional file 1: Appendix: Figure 1.** Random forest algorithm for prediction. **Figure 2.** Decision tree construction and prediction from randomforest algorithm. 

## Data Availability

In accordance with our Data Use Agreement with the Centers for Medicare and Medicaid Services, analytic datasets cannot be shared. However, aggregate data elements and coding algorithms will be made freely available upon request. Please contact the corresponding author to request aggregate data elements and coding algorithms.

## Abbreviations

CLI: Critical limb ischemia
COPD: Chronic obstructive pulmonary disease
DM: Diabetes Mellitus
FFS: Fee-for-Service
HbA1c: Hemoglobin A1c
ICD: International Classification of Diseases
ML: Machine Learning
MRD: Medicare Provider Analysis and Review
MBSF: Medicare Master Beneficiary Summary File
PAD: Peripheral Arterial Disease
RF: Random Forest
VI: Variable Importance
VQI: Vascular Quality Initiative
