# Supplementary material for: Using a cohort study of diabetes and peripheral artery disease to compare logistic regression and machine learning via random forest modeling
Source: BMC Med Res Methodol. 2022 Nov 23;22:300. doi: 10.1186/s12874-022-01774-8 (PMC9685056; doi:10.1186/s12874-022-01774-8)
Supplement: Supplementary file 1 — Additional file 1: Appendix: Figure 1. Random forest algorithm for prediction. Figure 2. Decision tree construction and prediction from randomforest algorithm. [file 12874_2022_1774_MOESM1_ESM.docx]

**Appendix**

**Figure 1: Random forest algorithm for prediction.**  *Ntree*, the number of trees to grow in the forest, is a parameter of the random forest algorithm. The *ntree* parameter is easy to tune, large numbers such as 500 and 1,000 are generally considered large enough when estimating the forest. The increase in predictive accuracy plateaus with an increase in *ntree*, with the timing of the plateau varying with each dataset considered. We selected an *ntree* of 500, an iteration after where our predictive accuracy plateaued as assessed by plotting the mean accuracy as *ntree* varied.

**
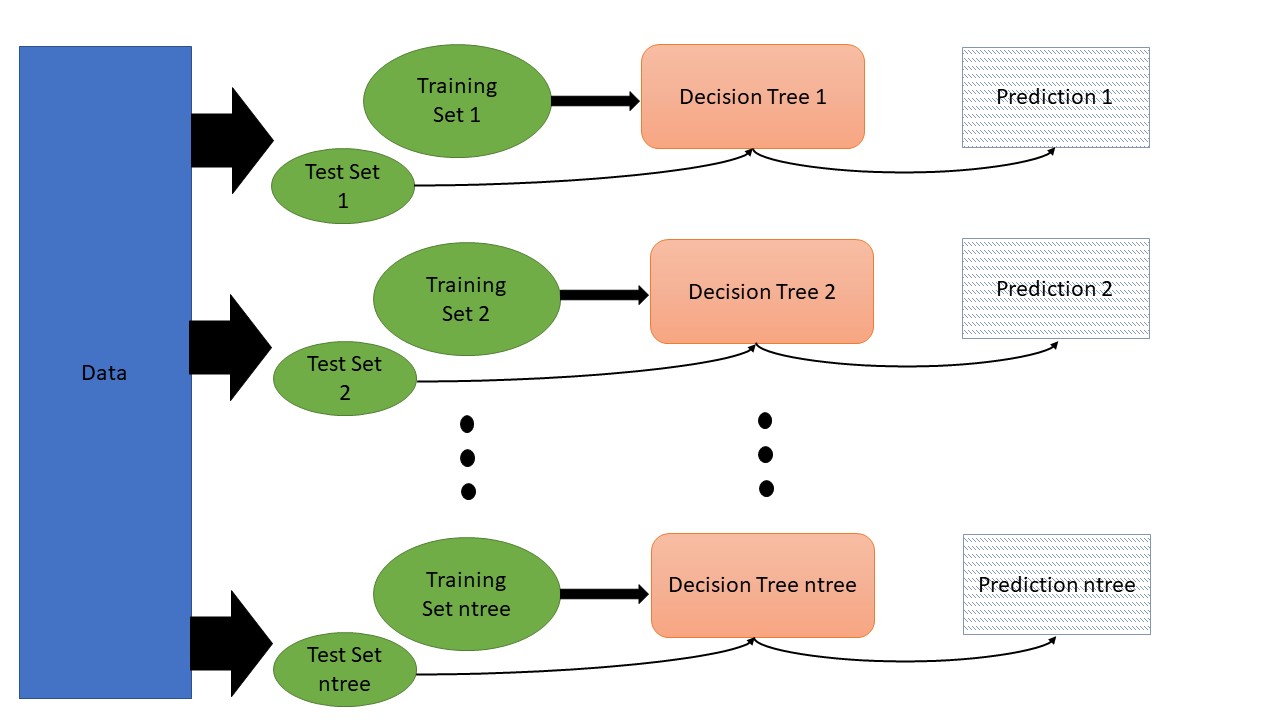
**

**Figure 2: Decision tree construction and prediction from random forest algorithm.** A second parameter of the random forest algorithm is *mtry*, the number of variables randomly sampled as candidates at each split in the tree. To select *mtry*, we fit a range of models for *mtry*=2 to the total number of covariates considered, 9, and selected the value which minimized the OOB error. In our data, *mtry=2* minimized the OOB error.

**
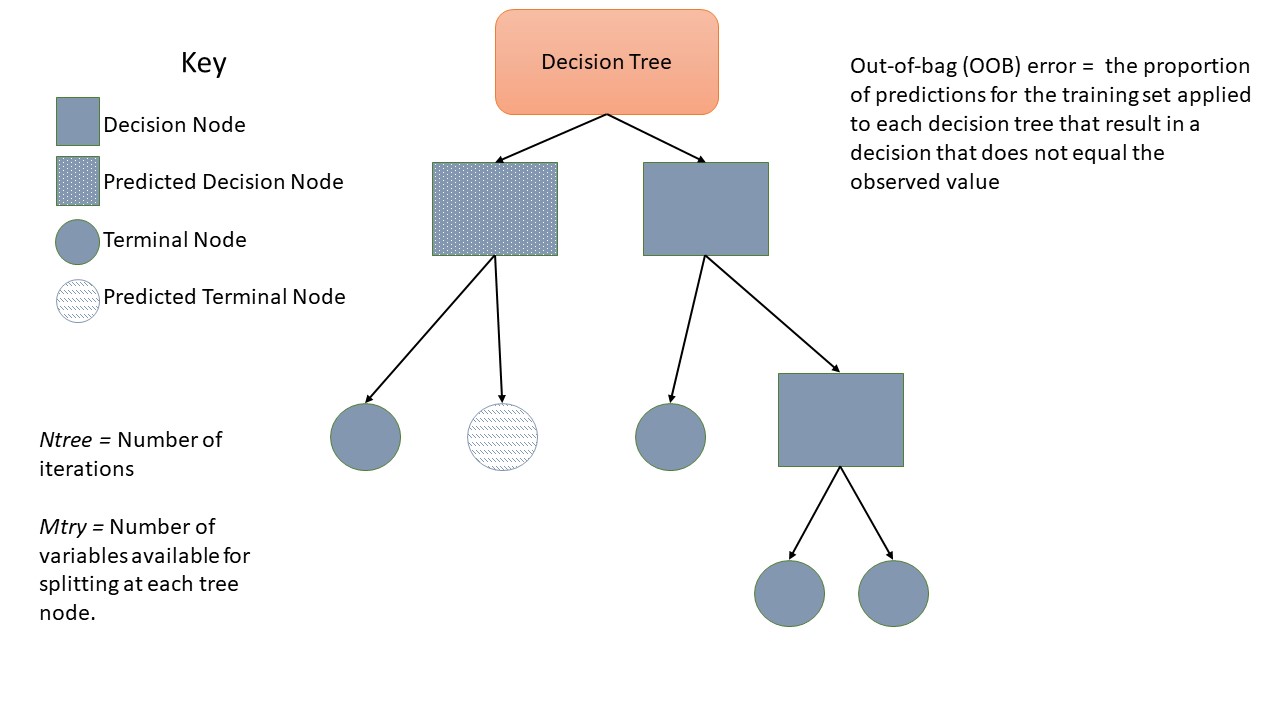
**
